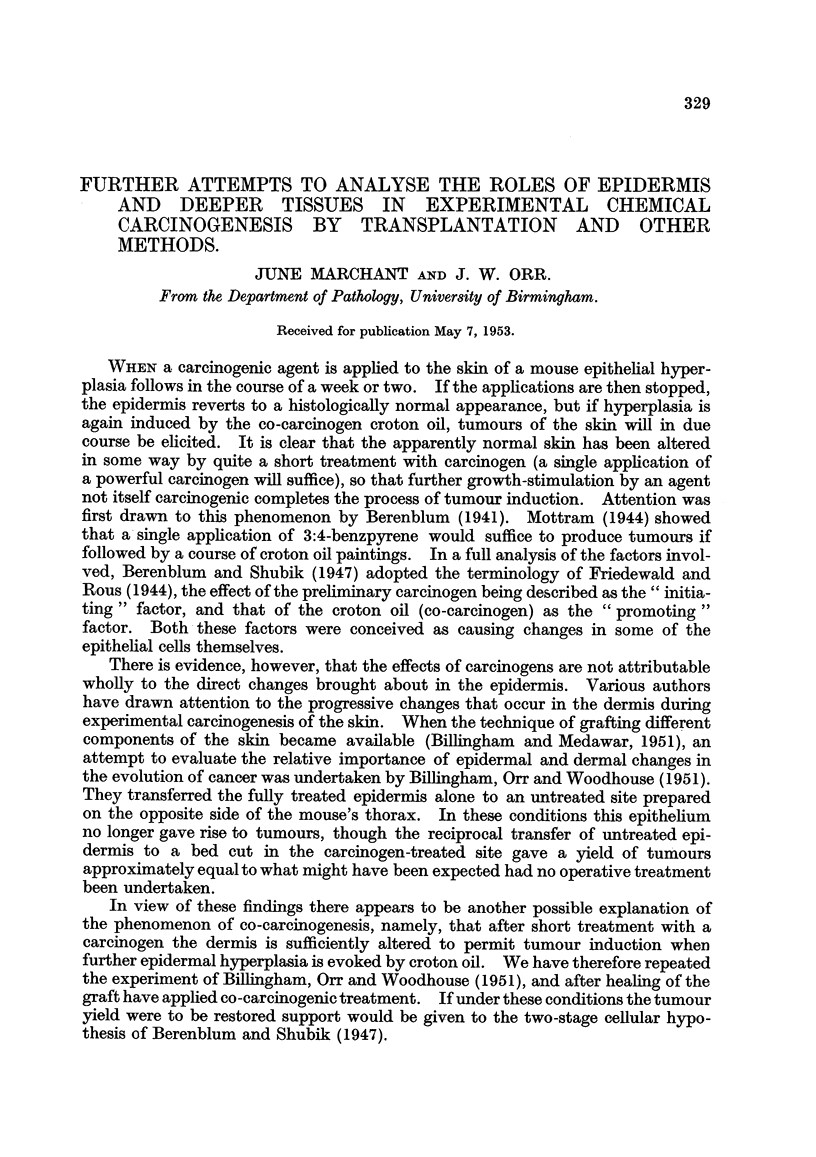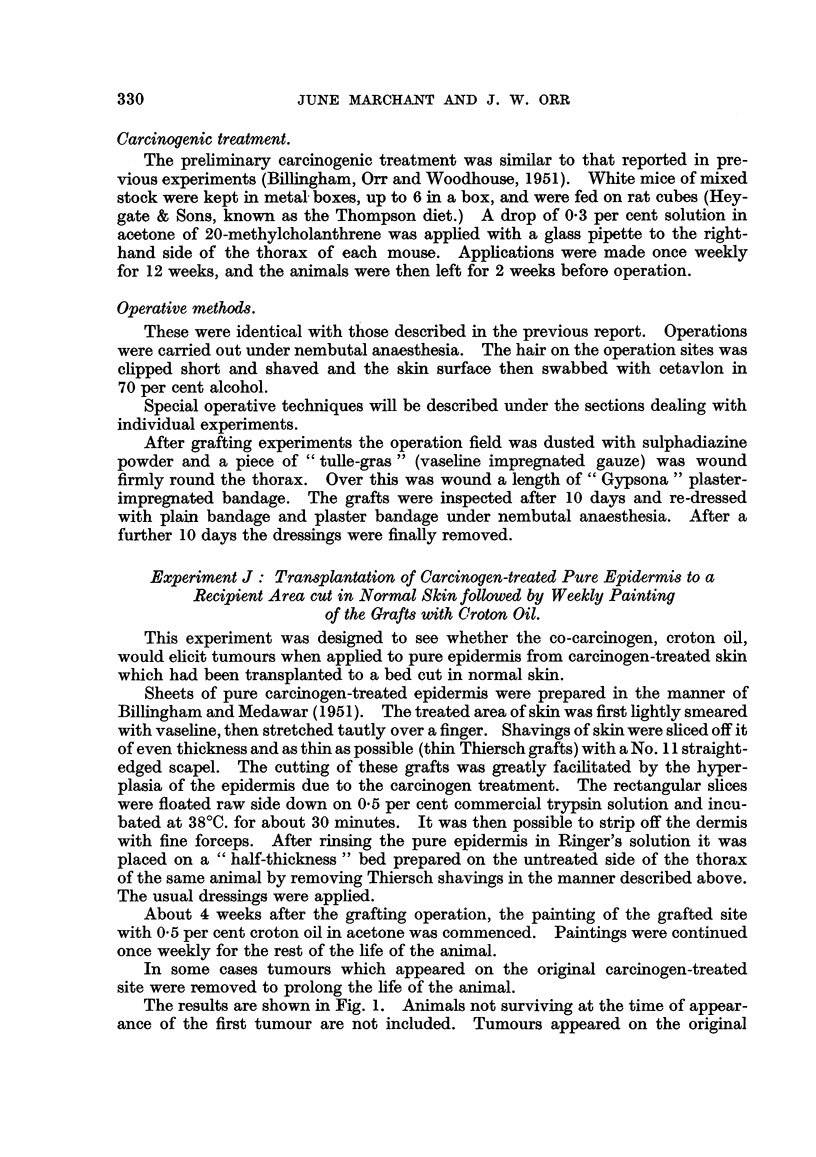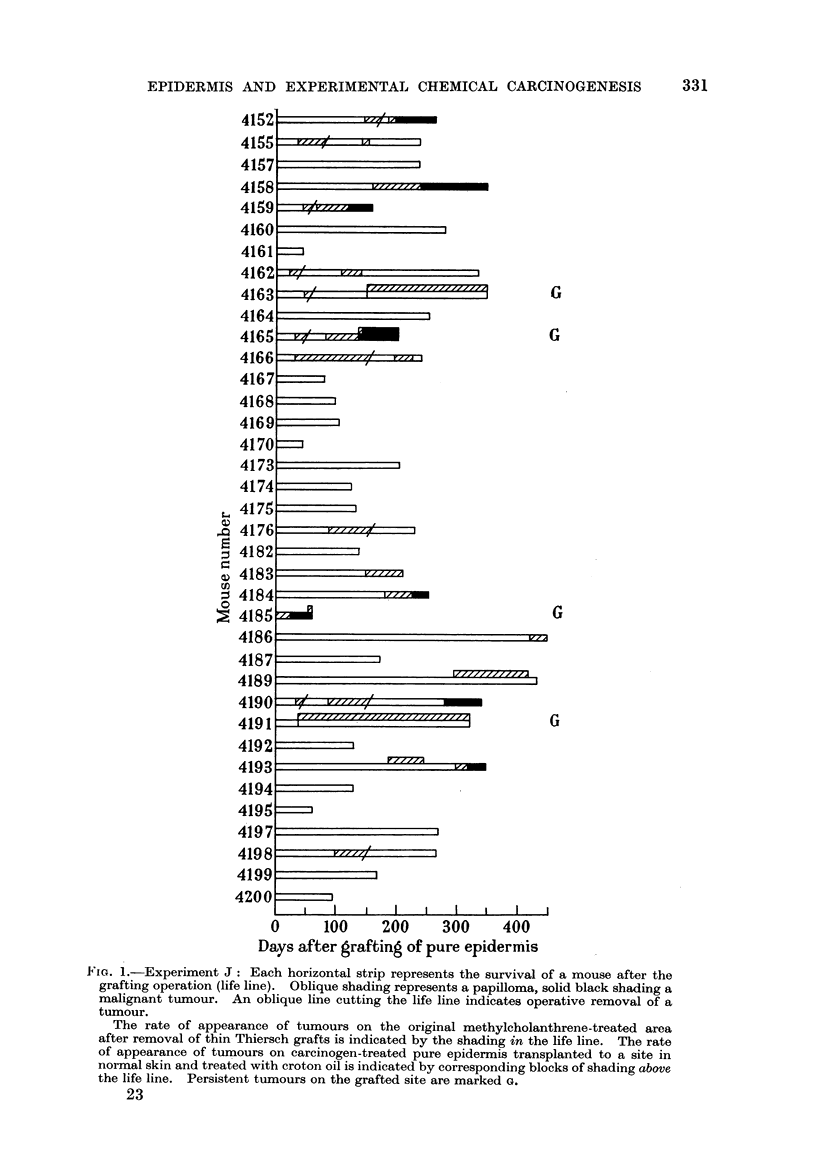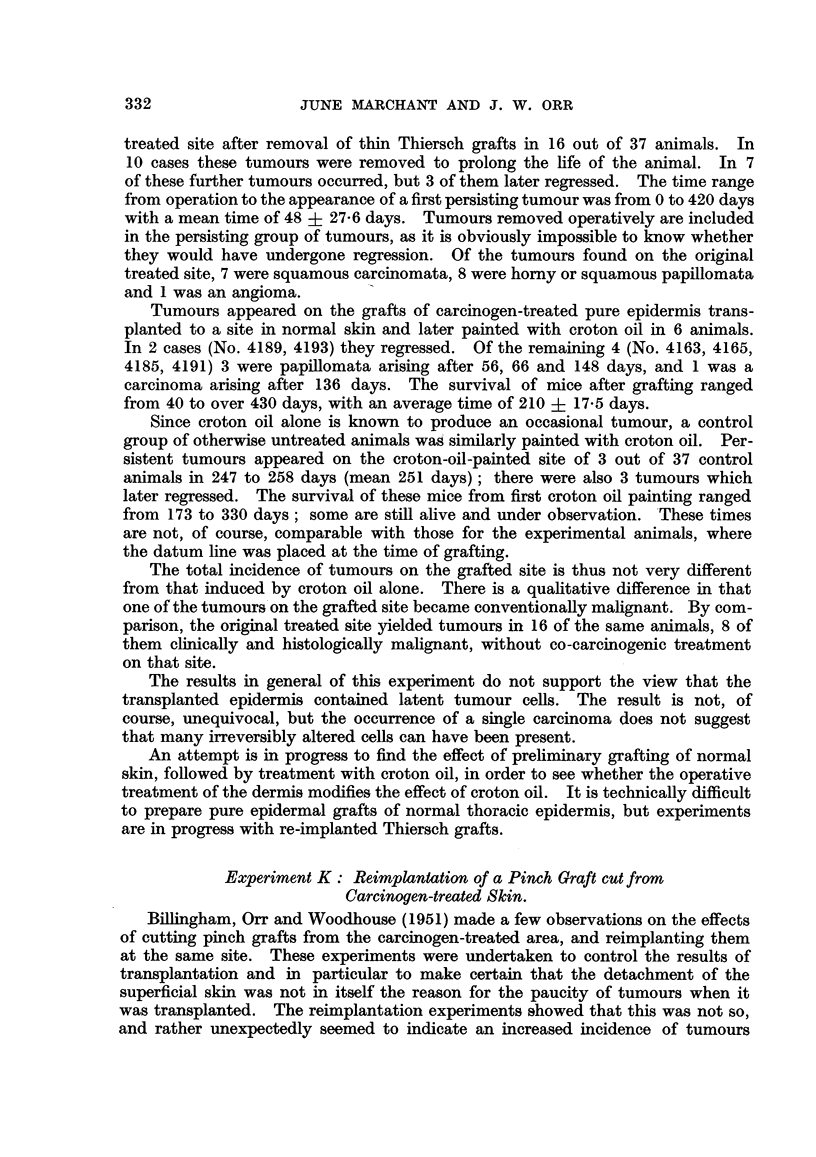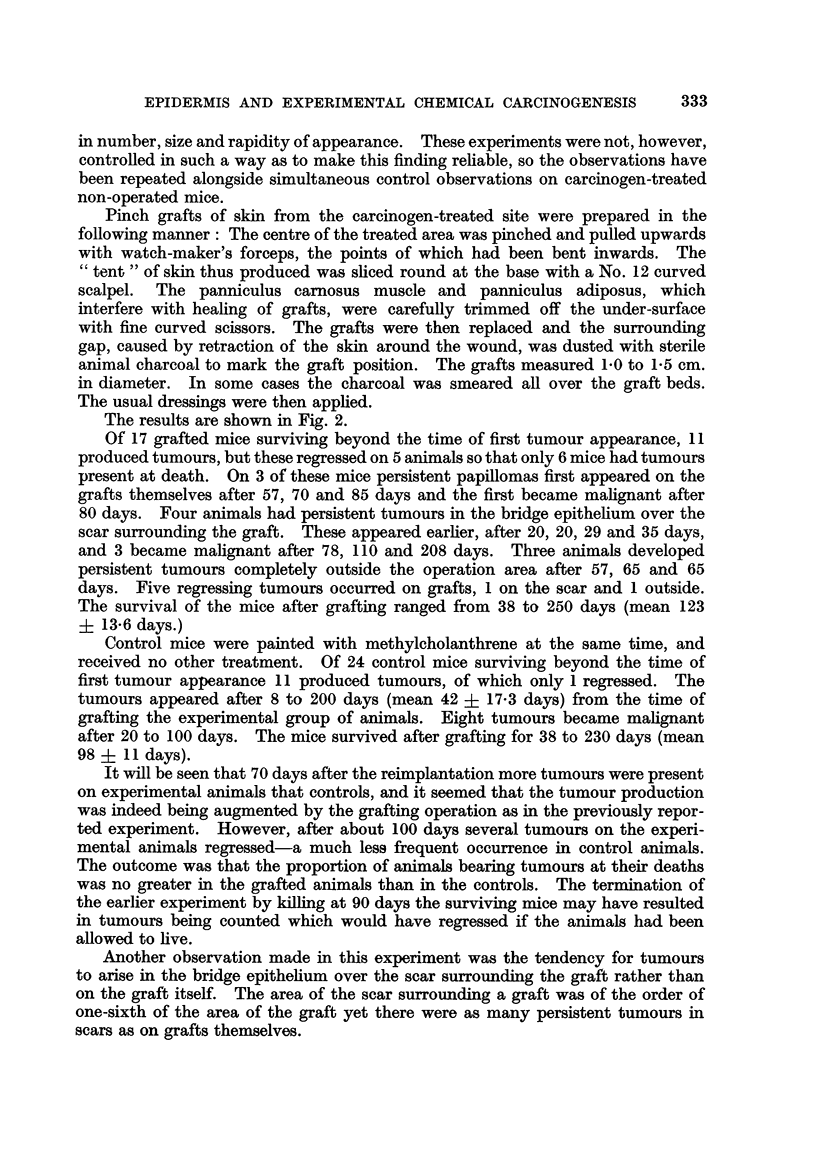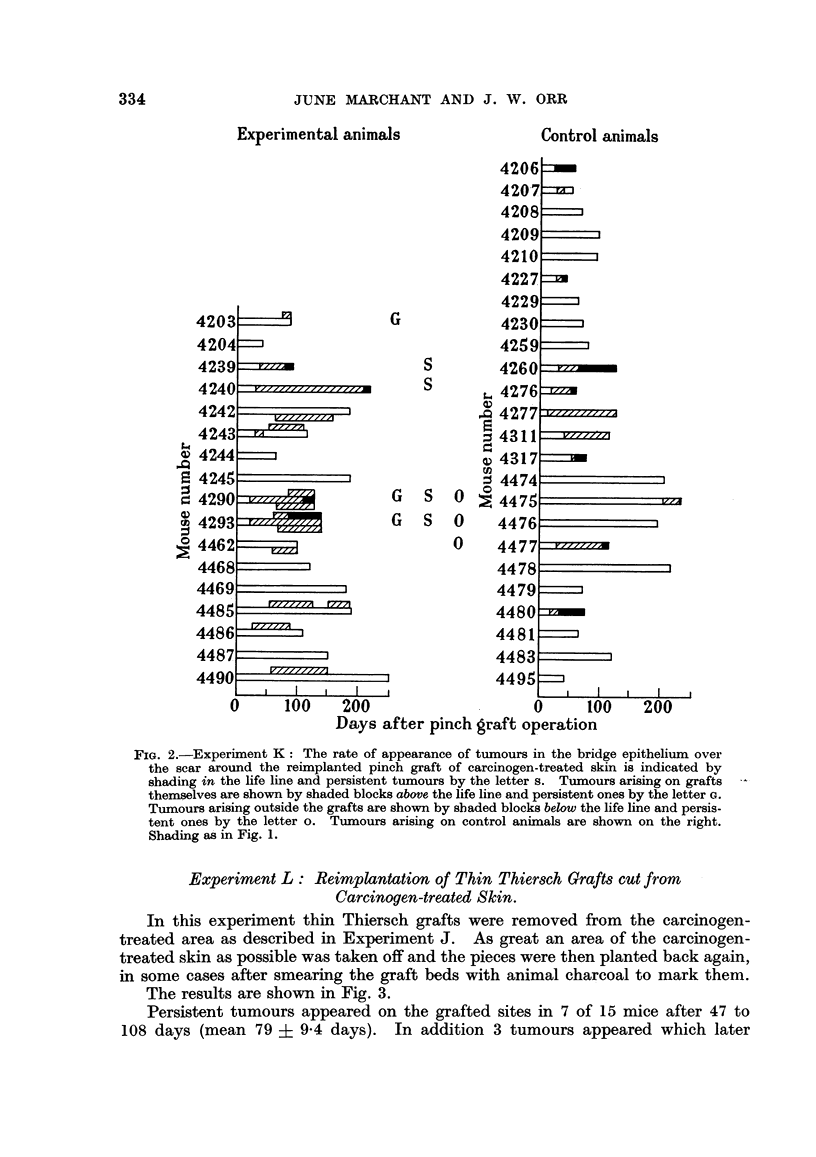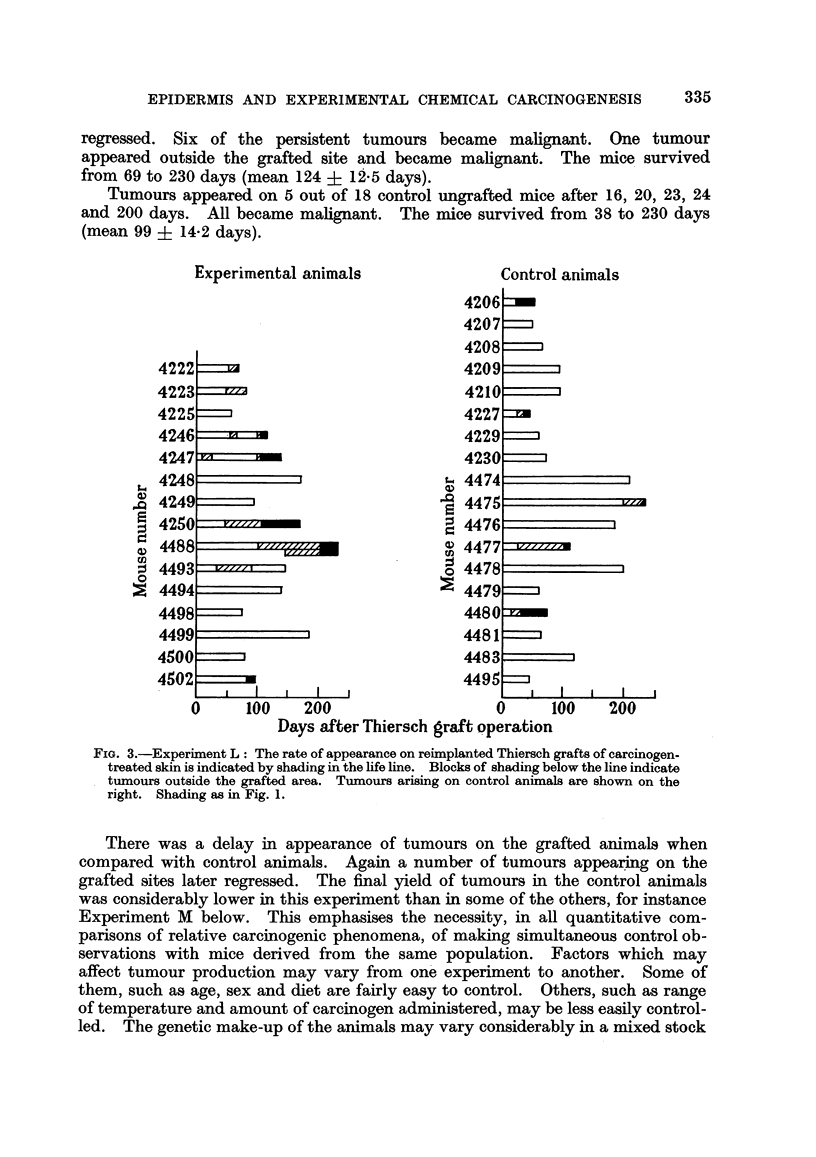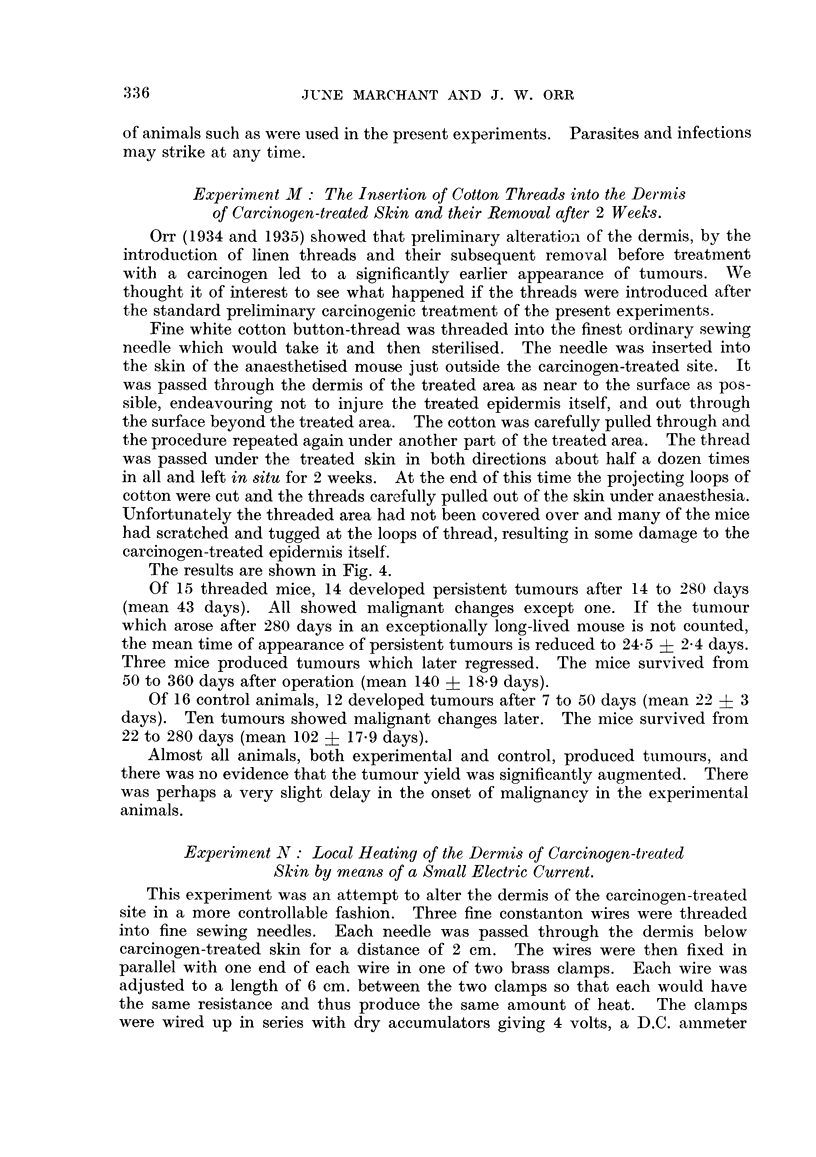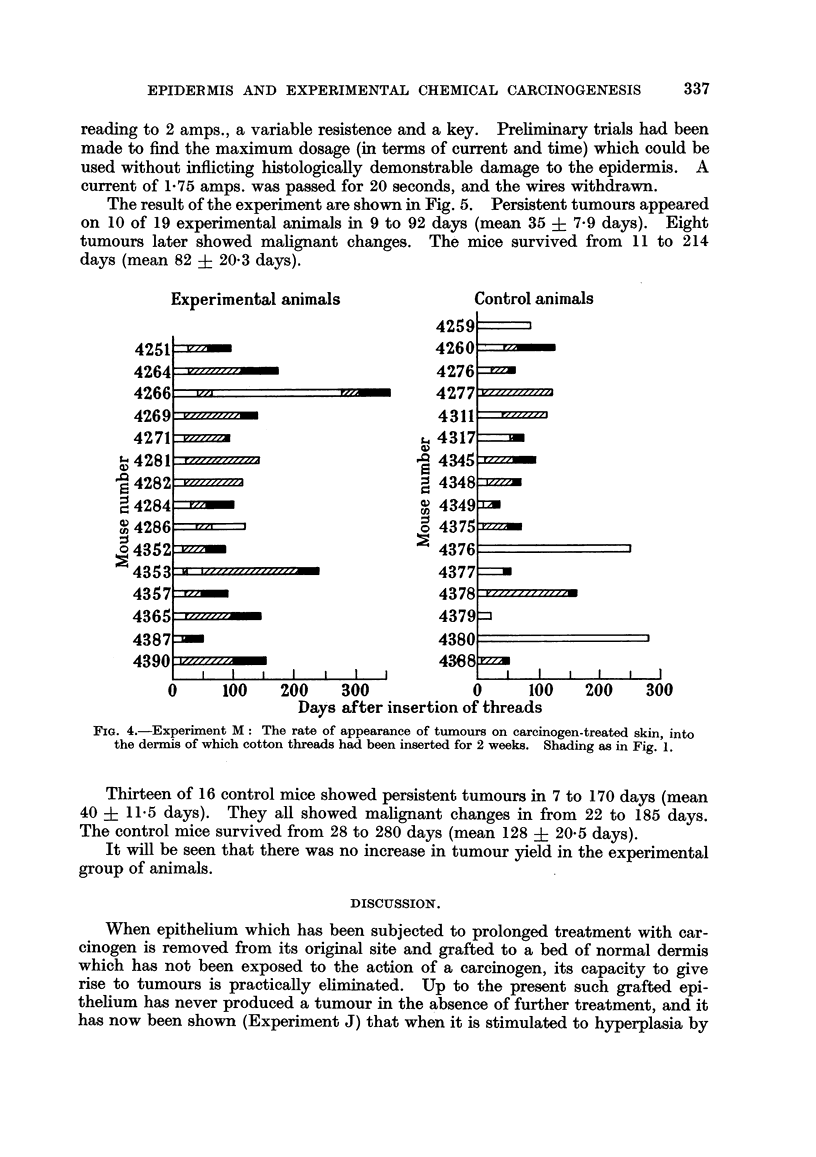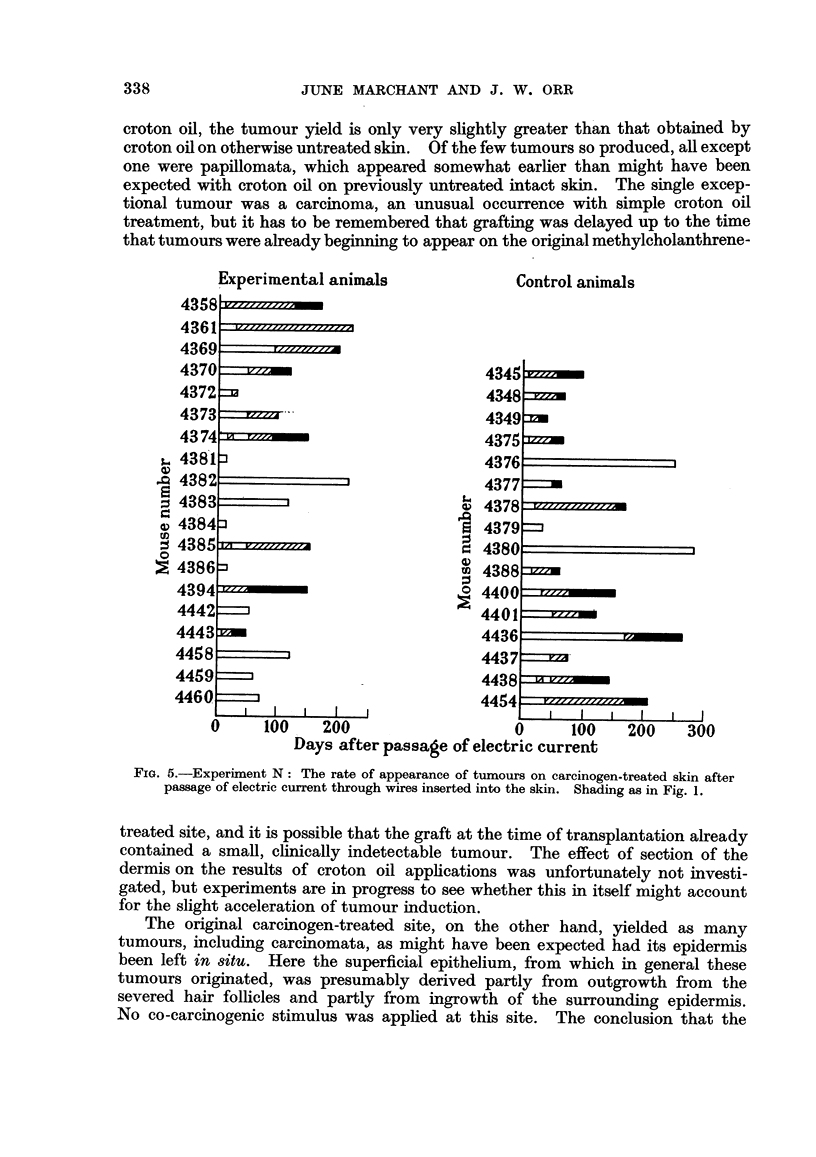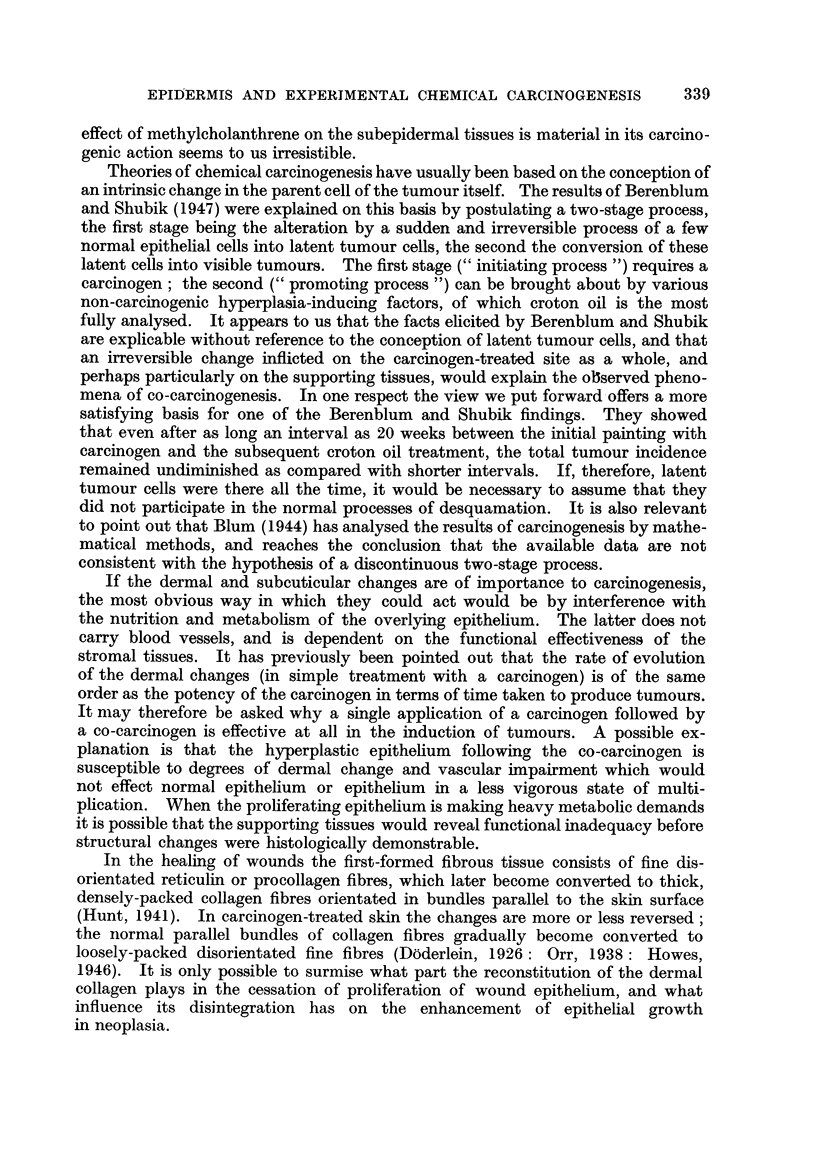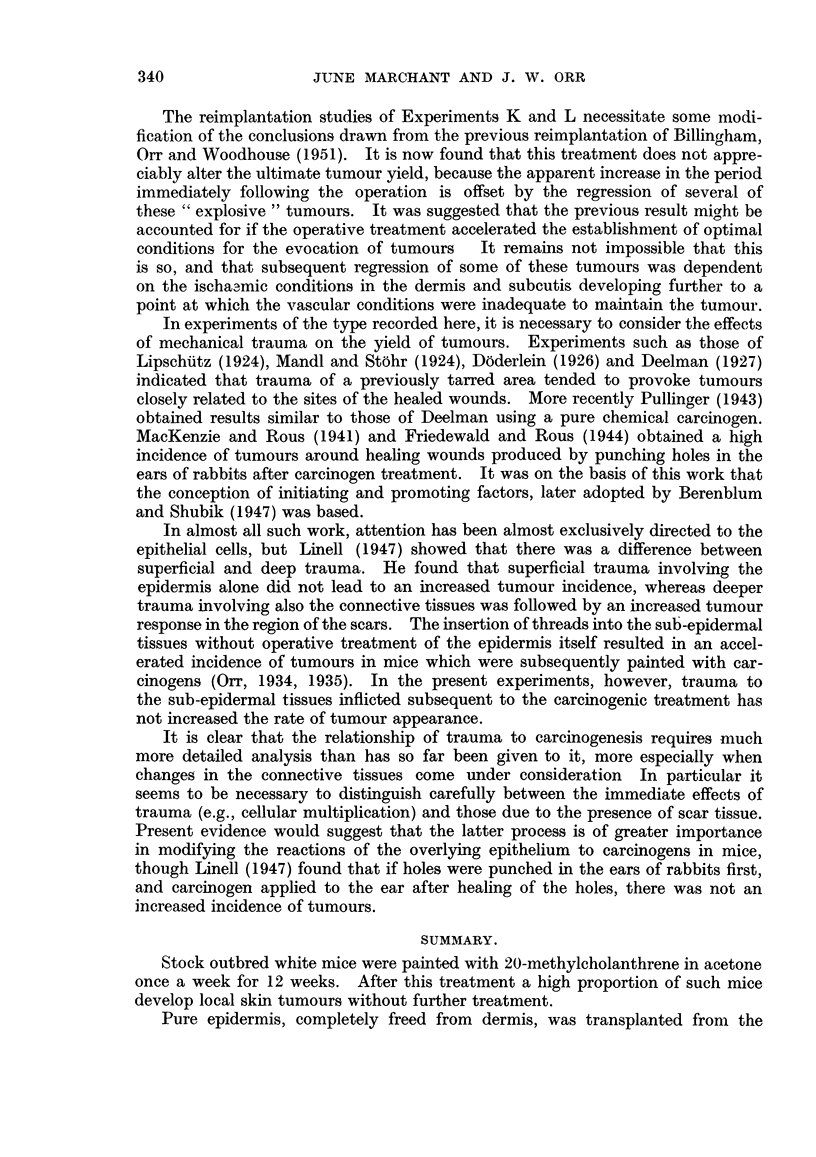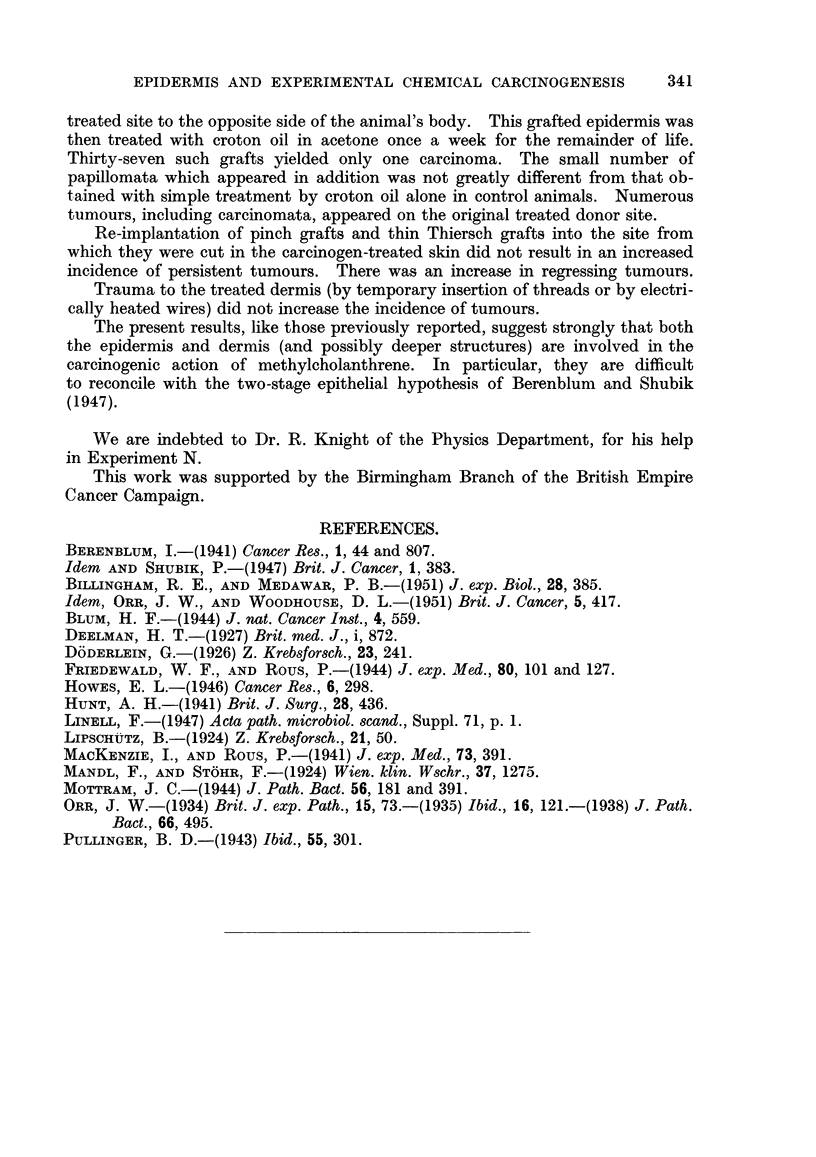# Further Attempts to Analyse the Roles of Epidermis and Deeper Tissues in Experimental Chemical Carcinogenesis by Transplantation and Other Methods

**DOI:** 10.1038/bjc.1953.31

**Published:** 1953-09

**Authors:** June Marchant, J. W. Orr


### FURTHER ATTEMPTS TO ANALYSE THE ROLES OF EPIDERMIS AND DEEPER TISSUES IN EXPERIMENTAL CHEMICAL CARCINOGENESIS BY TRANSPLANTATION AND OTHER METHODS.

JUNE MARCHANT AND J. W. ORR.

*From the Department of Pathology, University of Birmingham.*

Received for publication May 7, 1953.

WHEN a carcinogenic agent is applied to the skin of a mouse epithelial hyperplasia follows in the course of a week or two. If the applications are then stopped, the epidermis reverts to a histologically normal appearance, but if hyperplasia is again induced by the co-carcinogen croton oil, tumours of the skin will in due course be elicited. It is clear that the apparently normal skin has been altered in some way by quite a short treatment with carcinogen (a single application of a powerful carcinogen will suffice), so that further growth-stimulation by an agent not itself carcinogenic completes the process of tumour induction. Attention was first drawn to this phenomenon by Berenblum (1941). Mottram (1944) showed that a single application of 3:4-benzpyrene would suffice to produce tumours if followed by a course of croton oil paintings. In a full analysis of the factors involved, Berenblum and Shubik (1947) adopted the terminology of Friedewald and Rous (1944), the effect of the preliminary carcinogen being described as the " initiating " factor, and that of the croton oil (co-carcinogen) as the " promoting " factor. Both these factors were conceived as causing changes in some of the epithelial cells themselves.

There is evidence, however, that the effects of carcinogens are not attributable wholly to the direct changes brought about in the epidermis. Various authors have drawn attention to the progressive changes that occur in the dermis during experimental carcinogenesis of the skin. When the technique of grafting different components of the skin became available (Billingham and Medawar, 1951), an attempt to evaluate the relative importance of epidermal and dermal changes in the evolution of cancer was undertaken by Billingham, Orr and Woodhouse (1951). They transferred the fully treated epidermis alone to an untreated site prepared on the opposite side of the mouse's thorax. In these conditions this epithelium no longer gave rise to tumours, though the reciprocal transfer of untreated epidermis to a bed cut in the carcinogen-treated site gave a yield of tumours approximately equal to what might have been expected had no operative treatment been undertaken.

In view of these findings there appears to be another possible explanation of the phenomenon of co-carcinogenesis, namely, that after short treatment with a carcinogen the dermis is sufficiently altered to permit tumour induction when further epidermal hyperplasia is evoked by croton oil. We have therefore repeated the experiment of Billingham, Orr and Woodhouse (1951), and after healing of the graft have applied co-carcinogenic treatment. If under these conditions the tumour yield were to be restored support would be given to the two-stage cellular hypothesis of Berenblum and Shubik (1947).

*Carcinogenic treatment.*

The preliminary carcinogenic treatment was similar to that reported in previous experiments (Billingham, Orr and Woodhouse, 1951). White mice of mixed stock were kept in metal boxes, up to 6 in a box, and were fed on rat cubes (Heygate & Sons, known as the Thompson diet.) A drop of 0·3 per cent solution in acetone of 20-methylcholanthrene was applied with a glass pipette to the right-hand side of the thorax of each mouse. Applications were made once weekly for 12 weeks, and the animals were then left for 2 weeks before operation.

*Operative methods.*

These were identical with those described in the previous report. Operations were carried out under nembutal anaesthesia. The hair on the operation sites was clipped short and shaved and the skin surface then swabbed with cetavlon in 70 per cent alcohol.

Special operative techniques will be described under the sections dealing with individual experiments.

After grafting experiments the operation field was dusted with sulphadiazine powder and a piece of " tulle-gras " (vaseline impregnated gauze) was wound firmly round the thorax. Over this was wound a length of " Gypsona " plaster-impregnated bandage. The grafts were inspected after 10 days and re-dressed with plain bandage and plaster bandage under nembutal anaesthesia. After a further 10 days the dressings were finally removed.

### *Experiment J : Transplantation of Carcinogen-treated Pure Epidermis to a Recipient Area cut in Normal Skin followed by Weekly Painting of the Grafts with Croton Oil.*

This experiment was designed to see whether the co-carcinogen, croton oil, would elicit tumours when applied to pure epidermis from carcinogen-treated skin which had been transplanted to a bed cut in normal skin.

Sheets of pure carcinogen-treated epidermis were prepared in the manner of Billingham and Medawar (1951). The treated area of skin was first lightly smeared with vaseline, then stretched tautly over a finger. Shavings of skin were sliced off it of even thickness and as thin as possible (thin Thiersch grafts) with a No. 11 straight-edged scapel. The cutting of these grafts was greatly facilitated by the hyperplasia of the epidermis due to the carcinogen treatment. The rectangular slices were floated raw side down on 0·5 per cent commercial trypsin solution and incubated at 38°C. for about 30 minutes. It was then possible to strip off the dermis with fine forceps. After rinsing the pure epidermis in Ringer's solution it was placed on a " half-thickness " bed prepared on the untreated side of the thorax of the same animal by removing Thiersch shavings in the manner described above. The usual dressings were applied.

About 4 weeks after the grafting operation, the painting of the grafted site with 0·5 per cent croton oil in acetone was commenced. Paintings were continued once weekly for the rest of the life of the animal.

In some cases tumours which appeared on the original carcinogen-treated site were removed to prolong the life of the animal.

The results are shown in Fig. 1. Animals not surviving at the time of appearance of the first tumour are not included. Tumours appeared on the original

FIG. 1.—Experiment J : Each horizontal strip represents the survival of a mouse after the grafting operation (life line). Oblique shading represents a papilloma, solid black shading a malignant tumour. An oblique line cutting the life line indicates operative removal of a tumour.

The rate of appearance of tumours on the original methylcholanthrene-treated area after removal of thin Thiersch grafts is indicated by the shading *in* the life line. The rate of appearance of tumours on carcinogen-treated pure epidermis transplanted to a site in normal skin and treated with croton oil is indicated by corresponding blocks of shading *above* the life line. Persistent tumours on the grafted site are marked G.

treated site after removal of thin Thiersch grafts in 16 out of 37 animals. In 10 cases these tumours were removed to prolong the life of the animal. In 7 of these further tumours occurred, but 3 of them later regressed. The time range from operation to the appearance of a first persisting tumour was from 0 to 420 days with a mean time of 48 $\pm$ 27·6 days. Tumours removed operatively are included in the persisting group of tumours, as it is obviously impossible to know whether they would have undergone regression. Of the tumours found on the original treated site, 7 were squamous carcinomata, 8 were horny or squamous papillomata and 1 was an angioma.

Tumours appeared on the grafts of carcinogen-treated pure epidermis transplanted to a site in normal skin and later painted with croton oil in 6 animals. In 2 cases (No. 4189, 4193) they regressed. Of the remaining 4 (No. 4163, 4165, 4185, 4191) 3 were papillomata arising after 56, 66 and 148 days, and 1 was a carcinoma arising after 136 days. The survival of mice after grafting ranged from 40 to over 430 days, with an average time of 210 $\pm$ 17·5 days.

Since croton oil alone is known to produce an occasional tumour, a control group of otherwise untreated animals was similarly painted with croton oil. Persistent tumours appeared on the croton-oil-painted site of 3 out of 37 control animals in 247 to 258 days (mean 251 days); there were also 3 tumours which later regressed. The survival of these mice from first croton oil painting ranged from 173 to 330 days; some are still alive and under observation. These times are not, of course, comparable with those for the experimental animals, where the datum line was placed at the time of grafting.

The total incidence of tumours on the grafted site is thus not very different from that induced by croton oil alone. There is a qualitative difference in that one of the tumours on the grafted site became conventionally malignant. By comparison, the original treated site yielded tumours in 16 of the same animals, 8 of them clinically and histologically malignant, without co-carcinogenic treatment on that site.

The results in general of this experiment do not support the view that the transplanted epidermis contained latent tumour cells. The result is not, of course, unequivocal, but the occurrence of a single carcinoma does not suggest that many irreversibly altered cells can have been present.

An attempt is in progress to find the effect of preliminary grafting of normal skin, followed by treatment with croton oil, in order to see whether the operative treatment of the dermis modifies the effect of croton oil. It is technically difficult to prepare pure epidermal grafts of normal thoracic epidermis, but experiments are in progress with re-implanted Thiersch grafts.

*Experiment K: Reimplantation of a Pinch Graft cut from Carcinogen-treated Skin.*

Billingham, Orr and Woodhouse (1951) made a few observations on the effects of cutting pinch grafts from the carcinogen-treated area, and reimplanting them at the same site. These experiments were undertaken to control the results of transplantation and in particular to make certain that the detachment of the superficial skin was not in itself the reason for the paucity of tumours when it was transplanted. The reimplantation experiments showed that this was not so, and rather unexpectedly seemed to indicate an increased incidence of tumours in number, size and rapidity of appearance. These experiments were not, however, controlled in such a way as to make this finding reliable, so the observations have been repeated alongside simultaneous control observations on carcinogen-treated non-operated mice.

Pinch grafts of skin from the carcinogen-treated site were prepared in the following manner : The centre of the treated area was pinched and pulled upwards with watch-maker's forceps, the points of which had been bent inwards. The " tent " of skin thus produced was sliced round at the base with a No. 12 curved scalpel. The panniculus carnosus muscle and panniculus adiposus, which interfere with healing of grafts, were carefully trimmed off the under-surface with fine curved scissors. The grafts were then replaced and the surrounding gap, caused by retraction of the skin around the wound, was dusted with sterile animal charcoal to mark the graft position. The grafts measured 1·0 to 1·5 cm. in diameter. In some cases the charcoal was smeared all over the graft beds. The usual dressings were then applied.

The results are shown in Fig. 2.

Of 17 grafted mice surviving beyond the time of first tumour appearance, 11 produced tumours, but these regressed on 5 animals so that only 6 mice had tumours present at death. On 3 of these mice persistent papillomas first appeared on the grafts themselves after 57, 70 and 85 days and the first became malignant after 80 days. Four animals had persistent tumours in the bridge epithelium over the scar surrounding the graft. These appeared earlier, after 20, 20, 29 and 35 days, and 3 became malignant after 78, 110 and 208 days. Three animals developed persistent tumours completely outside the operation area after 57, 65 and 65 days. Five regressing tumours occurred on grafts, 1 on the scar and 1 outside. The survival of the mice after grafting ranged from 38 to 250 days (mean 123 $\pm$ 13·6 days.)

Control mice were painted with methylcholanthrene at the same time, and received no other treatment. Of 24 control mice surviving beyond the time of first tumour appearance 11 produced tumours, of which only 1 regressed. The tumours appeared after 8 to 200 days (mean 42 $\pm$ 17·3 days) from the time of grafting the experimental group of animals. Eight tumours became malignant after 20 to 100 days. The mice survived after grafting for 38 to 230 days (mean 98 $\pm$ 11 days).

It will be seen that 70 days after the reimplantation more tumours were present on experimental animals that controls, and it seemed that the tumour production was indeed being augmented by the grafting operation as in the previously reported experiment. However, after about 100 days several tumours on the experimental animals regressed—a much less frequent occurrence in control animals. The outcome was that the proportion of animals bearing tumours at their deaths was no greater in the grafted animals than in the controls. The termination of the earlier experiment by killing at 90 days the surviving mice may have resulted in tumours being counted which would have regressed if the animals had been allowed to live.

Another observation made in this experiment was the tendency for tumours to arise in the bridge epithelium over the scar surrounding the graft rather than on the graft itself. The area of the scar surrounding a graft was of the order of one-sixth of the area of the graft yet there were as many persistent tumours in scars as on grafts themselves.

FIG. 2.—Experiment K : The rate of appearance of tumours in the bridge epithelium over the scar around the reimplanted pinch graft of carcinogen-treated skin is indicated by shading *in* the life line and persistent tumours by the letter S. Tumours arising on grafts themselves are shown by shaded blocks *above* the life line and persistent ones by the letter G. Tumours arising outside the grafts are shown by shaded blocks *below* the life line and persistent ones by the letter O. Tumours arising on control animals are shown on the right. Shading as in Fig. 1.

### *Experiment L : Reimplantation of Thin Thiersch Grafts cut from Carcinogen-treated Skin.*

In this experiment thin Thiersch grafts were removed from the carcinogen-treated area as described in Experiment J. As great an area of the carcinogen-treated skin as possible was taken off and the pieces were then planted back again, in some cases after smearing the graft beds with animal charcoal to mark them. The results are shown in Fig. 3.

Persistent tumours appeared on the grafted sites in 7 of 15 mice after 47 to 108 days (mean 79 $\pm$ 9·4 days). In addition 3 tumours appeared which later regressed. Six of the persistent tumours became malignant. One tumour appeared outside the grafted site and became malignant. The mice survived from 69 to 230 days (mean 124 ± 12·5 days).

Tumours appeared on 5 out of 18 control ungrafted mice after 16, 20, 23, 24 and 200 days. All became malignant. The mice survived from 38 to 230 days (mean 99 ± 14·2 days).

FIG. 3.—Experiment L : The rate of appearance on reimplanted Thiersch grafts of carcinogen-treated skin is indicated by shading in the life line. Blocks of shading below the line indicate tumours outside the grafted area. Tumours arising on control animals are shown on the right. Shading as in Fig. 1.

There was a delay in appearance of tumours on the grafted animals when compared with control animals. Again a number of tumours appearing on the grafted sites later regressed. The final yield of tumours in the control animals was considerably lower in this experiment than in some of the others, for instance Experiment M below. This emphasises the necessity, in all quantitative comparisons of relative carcinogenic phenomena, of making simultaneous control observations with mice derived from the same population. Factors which may affect tumour production may vary from one experiment to another. Some of them, such as age, sex and diet are fairly easy to control. Others, such as range of temperature and amount of carcinogen administered, may be less easily controlled. The genetic make-up of the animals may vary considerably in a mixed stock of animals such as were used in the present experiments. Parasites and infections may strike at any time.

*Experiment M : The Insertion of Cotton Threads into the Dermis of Carcinogen-treated Skin and their Removal after 2 Weeks.*

Orr (1934 and 1935) showed that preliminary alteration of the dermis, by the introduction of linen threads and their subsequent removal before treatment with a carcinogen led to a significantly earlier appearance of tumours. We thought it of interest to see what happened if the threads were introduced after the standard preliminary carcinogenic treatment of the present experiments.

Fine white cotton button-thread was threaded into the finest ordinary sewing needle which would take it and then sterilised. The needle was inserted into the skin of the anaesthetised mouse just outside the carcinogen-treated site. It was passed through the dermis of the treated area as near to the surface as possible, endeavouring not to injure the treated epidermis itself, and out through the surface beyond the treated area. The cotton was carefully pulled through and the procedure repeated again under another part of the treated area. The thread was passed under the treated skin in both directions about half a dozen times in all and left *in situ* for 2 weeks. At the end of this time the projecting loops of cotton were cut and the threads carefully pulled out of the skin under anaesthesia. Unfortunately the threaded area had not been covered over and many of the mice had scratched and tugged at the loops of thread, resulting in some damage to the carcinogen-treated epidermis itself.

The results are shown in Fig. 4.

Of 15 threaded mice, 14 developed persistent tumours after 14 to 280 days (mean 43 days). All showed malignant changes except one. If the tumour which arose after 280 days in an exceptionally long-lived mouse is not counted, the mean time of appearance of persistent tumours is reduced to $24 \cdot 5 \pm 2 \cdot 4$ days. Three mice produced tumours which later regressed. The mice survived from 50 to 360 days after operation (mean $140 \pm 18 \cdot 9$ days).

Of 16 control animals, 12 developed tumours after 7 to 50 days (mean $22 \pm 3$ days). Ten tumours showed malignant changes later. The mice survived from 22 to 280 days (mean $102 \pm 17 \cdot 9$ days).

Almost all animals, both experimental and control, produced tumours, and there was no evidence that the tumour yield was significantly augmented. There was perhaps a very slight delay in the onset of malignancy in the experimental animals.

*Experiment N : Local Heating of the Dermis of Carcinogen-treated Skin by means of a Small Electric Current.*

This experiment was an attempt to alter the dermis of the carcinogen-treated site in a more controllable fashion. Three fine constanton wires were threaded into fine sewing needles. Each needle was passed through the dermis below carcinogen-treated skin for a distance of 2 cm. The wires were then fixed in parallel with one end of each wire in one of two brass clamps. Each wire was adjusted to a length of 6 cm. between the two clamps so that each would have the same resistance and thus produce the same amount of heat. The clamps were wired up in series with dry accumulators giving 4 volts, a D.C. ammeter reading to 2 amps., a variable resistence and a key. Preliminary trials had been made to find the maximum dosage (in terms of current and time) which could be used without inflicting histologically demonstrable damage to the epidermis. A current of 1·75 amps. was passed for 20 seconds, and the wires withdrawn.

The result of the experiment are shown in Fig. 5. Persistent tumours appeared on 10 of 19 experimental animals in 9 to 92 days (mean 35 $\pm$ 7·9 days). Eight tumours later showed malignant changes. The mice survived from 11 to 214 days (mean 82 $\pm$ 20·3 days).

FIG. 4.—Experiment M : The rate of appearance of tumours on carcinogen-treated skin, into the dermis of which cotton threads had been inserted for 2 weeks. Shading as in Fig. 1.

Thirteen of 16 control mice showed persistent tumours in 7 to 170 days (mean 40 $\pm$ 11·5 days). They all showed malignant changes in from 22 to 185 days. The control mice survived from 28 to 280 days (mean 128 $\pm$ 20·5 days).

It will be seen that there was no increase in tumour yield in the experimental group of animals.

### DISCUSSION.

When epithelium which has been subjected to prolonged treatment with carcinogen is removed from its original site and grafted to a bed of normal dermis which has not been exposed to the action of a carcinogen, its capacity to give rise to tumours is practically eliminated. Up to the present such grafted epithelium has never produced a tumour in the absence of further treatment, and it has now been shown (Experiment J) that when it is stimulated to hyperplasia by croton oil, the tumour yield is only very slightly greater than that obtained by croton oil on otherwise untreated skin. Of the few tumours so produced, all except one were papillomata, which appeared somewhat earlier than might have been expected with croton oil on previously untreated intact skin. The single exceptional tumour was a carcinoma, an unusual occurrence with simple croton oil treatment, but it has to be remembered that grafting was delayed up to the time that tumours were already beginning to appear on the original methylcholanthrene-

FIG. 5.—Experiment N : The rate of appearance of tumours on carcinogen-treated skin after passage of electric current through wires inserted into the skin. Shading as in Fig. 1.

treated site, and it is possible that the graft at the time of transplantation already contained a small, clinically indetectable tumour. The effect of section of the dermis on the results of croton oil applications was unfortunately not investigated, but experiments are in progress to see whether this in itself might account for the slight acceleration of tumour induction.

The original carcinogen-treated site, on the other hand, yielded as many tumours, including carcinomata, as might have been expected had its epidermis been left *in situ*. Here the superficial epithelium, from which in general these tumours originated, was presumably derived partly from outgrowth from the severed hair follicles and partly from ingrowth of the surrounding epidermis. No co-carcinogenic stimulus was applied at this site. The conclusion that the effect of methylcholanthrene on the subepidermal tissues is material in its carcinogenic action seems to us irresistible.

Theories of chemical carcinogenesis have usually been based on the conception of an intrinsic change in the parent cell of the tumour itself. The results of Berenblum and Shubik (1947) were explained on this basis by postulating a two-stage process, the first stage being the alteration by a sudden and irreversible process of a few normal epithelial cells into latent tumour cells, the second the conversion of these latent cells into visible tumours. The first stage (" initiating process ") requires a carcinogen ; the second (" promoting process ") can be brought about by various non-carcinogenic hyperplasia-inducing factors, of which croton oil is the most fully analysed. It appears to us that the facts elicited by Berenblum and Shubik are explicable without reference to the conception of latent tumour cells, and that an irreversible change inflicted on the carcinogen-treated site as a whole, and perhaps particularly on the supporting tissues, would explain the observed phenomena of co-carcinogenesis. In one respect the view we put forward offers a more satisfying basis for one of the Berenblum and Shubik findings. They showed that even after as long an interval as 20 weeks between the initial painting with carcinogen and the subsequent croton oil treatment, the total tumour incidence remained undiminished as compared with shorter intervals. If, therefore, latent tumour cells were there all the time, it would be necessary to assume that they did not participate in the normal processes of desquamation. It is also relevant to point out that Blum (1944) has analysed the results of carcinogenesis by mathematical methods, and reaches the conclusion that the available data are not consistent with the hypothesis of a discontinuous two-stage process.

If the dermal and subcuticular changes are of importance to carcinogenesis, the most obvious way in which they could act would be by interference with the nutrition and metabolism of the overlying epithelium. The latter does not carry blood vessels, and is dependent on the functional effectiveness of the stromal tissues. It has previously been pointed out that the rate of evolution of the dermal changes (in simple treatment with a carcinogen) is of the same order as the potency of the carcinogen in terms of time taken to produce tumours. It may therefore be asked why a single application of a carcinogen followed by a co-carcinogen is effective at all in the induction of tumours. A possible explanation is that the hyperplastic epithelium following the co-carcinogen is susceptible to degrees of dermal change and vascular impairment which would not effect normal epithelium or epithelium in a less vigorous state of multiplication. When the proliferating epithelium is making heavy metabolic demands it is possible that the supporting tissues would reveal functional inadequacy before structural changes were histologically demonstrable.

In the healing of wounds the first-formed fibrous tissue consists of fine disorientated reticulin or procollagen fibres, which later become converted to thick, densely-packed collagen fibres orientated in bundles parallel to the skin surface (Hunt, 1941). In carcinogen-treated skin the changes are more or less reversed ; the normal parallel bundles of collagen fibres gradually become converted to loosely-packed disorientated fine fibres (Döderlein, 1926 : Orr, 1938 : Howes, 1946). It is only possible to surmise what part the reconstitution of the dermal collagen plays in the cessation of proliferation of wound epithelium, and what influence its disintegration has on the enhancement of epithelial growth in neoplasia.

The reimplantation studies of Experiments K and L necessitate some modification of the conclusions drawn from the previous reimplantation of Billingham, Orr and Woodhouse (1951). It is now found that this treatment does not appreciably alter the ultimate tumour yield, because the apparent increase in the period immediately following the operation is offset by the regression of several of these " explosive " tumours. It was suggested that the previous result might be accounted for if the operative treatment accelerated the establishment of optimal conditions for the evocation of tumours It remains not impossible that this is so, and that subsequent regression of some of these tumours was dependent on the ischaemic conditions in the dermis and subcutis developing further to a point at which the vascular conditions were inadequate to maintain the tumour.

In experiments of the type recorded here, it is necessary to consider the effects of mechanical trauma on the yield of tumours. Experiments such as those of Lipschütz (1924), Mandl and Stöhr (1924), Döderlein (1926) and Deelman (1927) indicated that trauma of a previously tarred area tended to provoke tumours closely related to the sites of the healed wounds. More recently Pullinger (1943) obtained results similar to those of Deelman using a pure chemical carcinogen. MacKenzie and Rous (1941) and Friedewald and Rous (1944) obtained a high incidence of tumours around healing wounds produced by punching holes in the ears of rabbits after carcinogen treatment. It was on the basis of this work that the conception of initiating and promoting factors, later adopted by Berenblum and Shubik (1947) was based.

In almost all such work, attention has been almost exclusively directed to the epithelial cells, but Linell (1947) showed that there was a difference between superficial and deep trauma. He found that superficial trauma involving the epidermis alone did not lead to an increased tumour incidence, whereas deeper trauma involving also the connective tissues was followed by an increased tumour response in the region of the scars. The insertion of threads into the sub-epidermal tissues without operative treatment of the epidermis itself resulted in an accelerated incidence of tumours in mice which were subsequently painted with carcinogens (Orr, 1934, 1935). In the present experiments, however, trauma to the sub-epidermal tissues inflicted subsequent to the carcinogenic treatment has not increased the rate of tumour appearance.

It is clear that the relationship of trauma to carcinogenesis requires much more detailed analysis than has so far been given to it, more especially when changes in the connective tissues come under consideration In particular it seems to be necessary to distinguish carefully between the immediate effects of trauma (e.g., cellular multiplication) and those due to the presence of scar tissue. Present evidence would suggest that the latter process is of greater importance in modifying the reactions of the overlying epithelium to carcinogens in mice, though Linell (1947) found that if holes were punched in the ears of rabbits first, and carcinogen applied to the ear after healing of the holes, there was not an increased incidence of tumours.

### SUMMARY.

Stock outbred white mice were painted with 20-methylcholanthrene in acetone once a week for 12 weeks. After this treatment a high proportion of such mice develop local skin tumours without further treatment.

Pure epidermis, completely freed from dermis, was transplanted from the treated site to the opposite side of the animal's body. This grafted epidermis was then treated with croton oil in acetone once a week for the remainder of life. Thirty-seven such grafts yielded only one carcinoma. The small number of papillomata which appeared in addition was not greatly different from that obtained with simple treatment by croton oil alone in control animals. Numerous tumours, including carcinomata, appeared on the original treated donor site.

Re-implantation of pinch grafts and thin Thiersch grafts into the site from which they were cut in the carcinogen-treated skin did not result in an increased incidence of persistent tumours. There was an increase in regressing tumours.

Trauma to the treated dermis (by temporary insertion of threads or by electrically heated wires) did not increase the incidence of tumours.

The present results, like those previously reported, suggest strongly that both the epidermis and dermis (and possibly deeper structures) are involved in the carcinogenic action of methylcholanthrene. In particular, they are difficult to reconcile with the two-stage epithelial hypothesis of Berenblum and Shubik (1947).

We are indebted to Dr. R. Knight of the Physics Department, for his help in Experiment N.

This work was supported by the Birmingham Branch of the British Empire Cancer Campaign.

### REFERENCES.

BERENBLUM, I.—(1941) *Cancer Res.*, **1**, 44 and 807.
*Idem* AND SHUBIK, P.—(1947) *Brit. J. Cancer*, **1**, 383.
BILLINGHAM, R. E., AND MEDAWAR, P. B.—(1951) *J. exp. Biol.*, **28**, 385.
*Idem*, ORR, J. W., AND WOODHOUSE, D. L.—(1951) *Brit. J. Cancer*, **5**, 417.
BLUM, H. F.—(1944) *J. nat. Cancer Inst.*, **4**, 559.
DEELMAN, H. T.—(1927) *Brit. med. J.*, i, 872.
DÖDERLEIN, G.—(1926) *Z. Krebsforsch.*, **23**, 241.
FRIEDEWALD, W. F., AND ROUS, P.—(1944) *J. exp. Med.*, **80**, 101 and 127.
HOWES, E. L.—(1946) *Cancer Res.*, **6**, 298.
HUNT, A. H.—(1941) *Brit. J. Surg.*, **28**, 436.
LINELL, F.—(1947) *Acta path. microbiol. scand.*, Suppl. 71, p. 1.
LIPSCHÜTZ, B.—(1924) *Z. Krebsforsch.*, **21**, 50.
MACKENZIE, I., AND ROUS, P.—(1941) *J. exp. Med.*, **73**, 391.
MANDL, F., AND STÖHR, F.—(1924) *Wien. klin. Wschr.*, **37**, 1275.
MOTTRAM, J. C.—(1944) *J. Path. Bact.* **56**, 181 and 391.
ORR, J. W.—(1934) *Brit. J. exp. Path.*, **15**, 73.—(1935) *Ibid.*, **16**, 121.—(1938) *J. Path. Bact.*, **66**, 495.
PULLINGER, B. D.—(1943) *Ibid.*, **55**, 301.